# Multifunctional Sensor for Strain, Pressure, and UV Light Detections Using Polyaniline and ZnO Nanostructures on a Flexible Substrate

**DOI:** 10.3390/polym17131825

**Published:** 2025-06-30

**Authors:** Seung-Woo Lee, Ju-Seong Lee, Hyeon-Wook Yu, Tae-Hee Kim, Hyun-Seok Kim

**Affiliations:** Division of Electronics and Electrical Engineering, Dongguk University, Seoul 04620, Republic of Korea; dltmddn0226@dgu.ac.kr (S.-W.L.); juseong1806@dongguk.edu (J.-S.L.); jejuyhw@dgu.ac.kr (H.-W.Y.); 2022111798@dgu.ac.kr (T.-H.K.)

**Keywords:** multifunctional sensor, flexible device, polyaniline (PANI), zinc oxide (ZnO), strain, pressure, ultraviolet (UV)

## Abstract

Wearable sensors have rapidly advanced, enabling applications such as human activity monitoring, electronic skin, and biomimetic robotics. To meet the growing demands of these applications, multifunctional sensing has become essential for wearable devices. However, most existing studies predominantly focus on enhancing single-function sensing capabilities. This study introduces a multifunctional sensor that combines high stretchability for strain and pressure detection with ultraviolet (UV) sensing capability. To achieve simultaneous detection of strain, pressure, and UV light, a multi-sensing approach was employed: a capacitive method for strain and pressure detections and a resistive method utilizing a pn-heterojunction diode for UV detection. In the capacitive method, polyaniline (PANI) served as parallel-plate electrodes, while silicon-based elastomer acted as the dielectric layer. This configuration enabled up to 100% elongation and enhanced operational stability through encapsulation. The sensor demonstrated a strong linear relationship between capacitance value changes reasonably based on the area of PANI, and showed a good linearity with an R-squared value of 0.9918. It also detected pressure across a wide range, from low (0.4 kPa) to high (9.4 kPa). Furthermore, for wearable applications, the sensor reliably captured capacitance variations during finger bending at different angles. For UV detection, a pn-heterojunction diode composed of p-type silicon and n-type zinc oxide nanorods exhibited a rapid response time of 6.1 s and an on/off ratio of 13.8 at −10 V. Durability under 100% tensile strain was confirmed through Von Mises stress calculations using finite element modeling. Overall, this multifunctional sensor offers significant potential for a variety of applications, including human motion detection, wearable technology, and robotics.

## 1. Introduction

For the next generation of wearable devices, multifunctional sensors capable of detecting various environmental stimuli such as strain, pressure, ultraviolet (UV) radiation, and temperature are essential [1,2,3,4]. Such sensors must be inherently flexible to accommodate deformations caused by human skin movement and must exhibit high stability during repetitive use [5,6]. Integrating strain and pressure sensing with ultraviolet monitoring enables concurrent quantification of skin deformation, medical tracking, and incident UV dose, supporting applications that range from outdoor-exercise analytics to rehabilitation feedback [7]. As a result, significant research efforts are focused on utilizing various one-dimensional nanomaterials to enhance sensor performance [8,9].

Among these materials, polyaniline (PANI) stands out due to its high conductivity, cost-effectiveness, and environmental stability. PANI can detect a range of stimuli, including gas, deformation-induced resistance changes, and temperature, making it widely adopted in flexible sensors. Derived from its π-conjugated structure, PANI easily loses electrons to dopants, forming a delocalized charge and exhibiting p-type semiconductor characteristics. Its work function, which ranges from 4.2 to 4.7 eV depending on the synthesis method, significantly influences its properties. When synthesized via electrochemical methods, PANI nanofibers (PANI NFs) demonstrate enhanced performance under tension due to their island-bridge structure, enabling the creation of tensile sensors with high elasticity and sensitivity [10,11,12].

Zinc oxide (ZnO), with its wide and direct bandgap of 3.37 eV, is commonly used in UV detectors. Its non-toxicity and biocompatibility make it well-suited for wearable sensors. ZnO functions as an n-type semiconductor, exhibiting high conductivity due to oxygen vacancies within its hexagonal wurtzite structure. A p-type silicon (p-Si)/n-type ZnO (n-ZnO) heterojunction photodetector leverages electron–hole pair (EHP) generation under the reverse bias condition to facilitate current flow. Among ZnO nanostructures, ZnO nanorods (ZnO NRs) are particularly effective for photodetection, owing to their high surface-to-volume ratio, which enhances UV sensitivity [13,14,15].

Practical wearable sensors require materials with properties similar to human skin, particularly flexibility and adaptability. Silicon-based elastomers, such as Ecoflex and polydimethylsiloxane (PDMS), are widely used in sensor fabrication due to their biocompatibility and long-term stability. Ecoflex, in particular, is environmentally stable and suitable for long-term sensing applications, ensuring comfort and reliability when used as a skin-mounted device [16]. Additionally, Ecoflex demonstrates excellent performance in strain sensors, offering flexibility, linearity, low hysteresis, and mechanical stability [17].

Research on sensors utilizing PANI and ZnO has primarily focused on enhancing single-sensing capabilities using one-dimensional nanomaterials or various composites [18,19,20,21]. While resistive strain sensors are commonly used due to their simple structure and manufacturing process, they often suffer from nonlinearity and severe hysteresis [22,23]. Resistance variations in these sensors can be influenced not only by structural deformations but also by changes in material conductivity, complicating accurate measurements [24]. For instance, research has shown that resistive strain sensors using PANI face limitations in stability under high tensile strain, with maximum strain rates of only 40% [25]. Moreover, strain signals in resistive strain sensors are often coupled with pressure signals, leading to further challenges in achieving precise measurements [26].

In contrast, capacitive strain sensors, which rely on geometric changes in the dielectric material and electrodes, offer advantages such as improved stretchability, linearity, and reduced hysteresis [22]. While capacitive sensors in most studies utilize liquid metal electrodes, research on PANI-based capacitive sensors remains unexplored [27,28]. Additionally, no prior studies have combined capacitive and resistive sensing methods on the same substrate. Implementing both sensing methods on a shared electrode poses challenges, such as signal interference caused by overlapping AC voltage in capacitive sensors and DC voltage in resistive sensors [29].

This study addresses these challenges by separately implementing two sensing components—capacitive and resistive—on the same flexible substrate, effectively eliminating signal interference. By leveraging the properties of PANI NFs and ZnO NRs alongside the flexibility of Ecoflex, we present a multifunctional sensor that achieves superior performance in both sensing modalities.

## 2. Materials and Methods

### 2.1. Materials

Aniline (≥99.5%, 62-53-3), hydrochloric acid (≥37%, 7647-01-0), sulfuric acid (≥98%, 7664-93-9), n-propanol (71-23-8), zinc acetate dihydrate (≥98%, 5970-45-6), methenamine (100-97-0), and zinc nitrate hexahydrate (≥98%, 10196-18-6) were purchased from Sigma-Aldrich (Seoul, Korea). Silver pellets (≥99.99%), silver paste (7440-22), ITO-coated glass (0.7 mm thickness, 20 Ω/cm^2^), PEN film (0.2 mm thickness), and p-type Si wafers (500 μm thickness, 10 Ω·cm, doped with boron) were sourced from iTASCO (Seoul, Republic of Korea). A graphite rod was acquired from QRINS (Seoul, Korea). PDMS base and curing agent (Sylgard 184) were obtained from Dow Corning (Midland, MI, USA). Ecoflex 00-10 was purchased from Smooth-On (Macungie, PA, USA). Copper foil tape (100%, 0.005 Ω·cm) was obtained from 3M (Seoul, Republic of Korea). All materials were used as received without further purification.

### 2.2. Characterization

The surface morphology of ZnO NRs and PANI NFs was analyzed using field emission scanning electron microscopy (FE-SEM, TESCAN, Brno, Czech Republic). A TEC-01 force analyzer (DigiTech, Osaka, Japan) was used for tensile sensing characterization. Capacitance measurements and AC voltage supply were performed using a 41100 LCR meter (Wayne Kerr Electronics, Cavendish Square, London, UK). A model 2450 source measure unit (Keithley, Cleveland, OH, USA) was used to supply DC voltages in the capacitive sensor and to measure the current of the photodetector. UV light was supplied by a 70714 sample bias amplifier, SR810 DSP lock-in amplifier (Stanford Research Systems, Sunnyvale, CA, USA), CS260-USB-1-MC-D UV-Vis monochromator, OPS-A500 arc lamp power supply, USFW-100 universal filter wheel, 66902 arc lamp housing, and 3502 optical choppers (Newport, Irvine, CA, USA).

### 2.3. Design of Multifunctional Sensor

**Figure 1:**
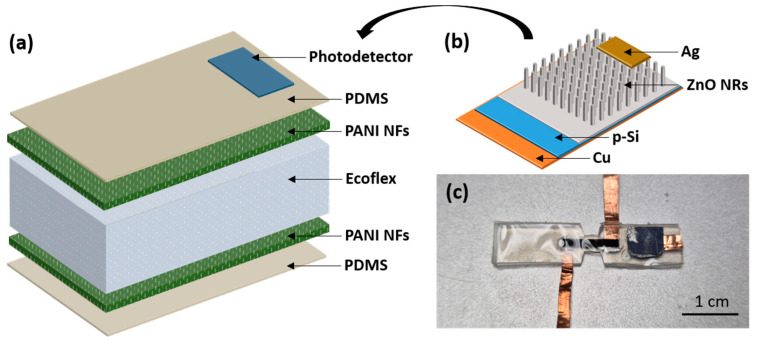
(**a**) Schematic illustration of multifunctional sensor; (**b**) schematic diagram of photodetector; (**c**) photograph of fabricated multifunctional sensor.

A multifunctional sensor capable of detecting strain, pressure, and UV light was developed for use in electronic skin (e-skin) applications. Transparent and flexible sensors are essential for such applications. To fabricate the sensor, we integrated a tensile sensor and a photodetector onto a single flexible substrate. The tensile sensor employs a capacitive sensing method, while UV detection is achieved using a photodetector based on a pn-heterojunction diode. The tensile sensor consists of a thin PDMS layer for encapsulation, PANI NFs as the electrodes, and Ecoflex as the dielectric layer of the capacitor [Figure 1a]. Ecoflex, with its exceptional elongation, was chosen for the dielectric layer due to its suitability for strain sensor applications. PDMS, with Young’s modulus ranging from 0.1 to 1 MPa, is ideal for use in skin-attached sensors. To further stabilize the sensor, copper tape was embedded by encapsulation with silver paste to reduce contact resistance. Both the strain sensor and photodetector formed ohmic contacts, ensuring stable performance [30,31].

The photodetector comprises a pn-heterojunction structure of p-Si and n-ZnO [Figure 1b]. By bonding these components together, we created a multifunctional sensor that can detect both strain, pressure, and UV light on the same substrate [Figure 1c]. Using this photodetector, we significantly improved the UV detection response time, achieving a performance increase of approximately 14.6 times compared to conventional devices [25].

However, bonding a flat Si wafer to a stretchable substrate can limit the flexibility of the sensor. To address this issue, we used finite element modeling simulations (COMSOL Multiphysics, Burlington, MA, USA) to calculate the Von Mises stress on the Si wafer during stretching, preventing potential fracture. Adhesion between the PDMS and Si wafer can also be problematic. To improve the adhesion, we treated the PDMS and copper tape surfaces with O_2_ plasma treatment (100 W RF power, 50 sccm oxygen flow, 120 mtorr, 300 s), which modified their surface characteristics to enhance bonding. In addition, to reinforce interfacial adhesion, we applied an additional Ecoflex over molding layer, which effectively suppresses peel-off even under repeated mechanical deformation.

Furthermore, supplying the capacitive element with an AC excitation (0–2 V, 1 MHz) while biasing the photodetector with a DC voltage through separate electrodes completely eliminates cross-talk between two sensing channels. Consequently, this decoupling strategy enables the seamless integration of multi-sensing modalities on a single substrate without mutual interference.

### 2.4. Fabrication of Capacitive Sensor Using PANI

**Figure 2:**
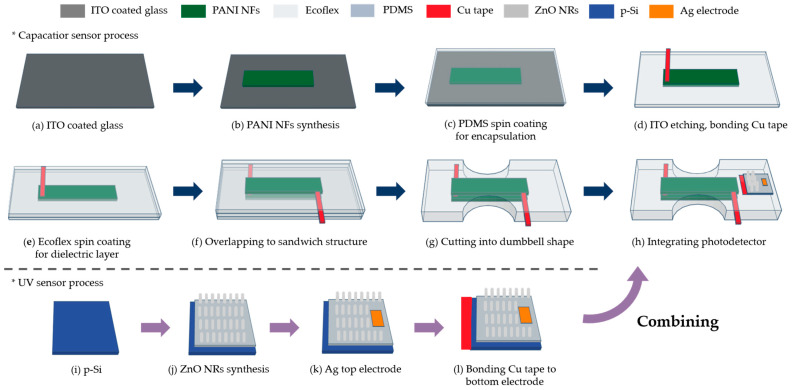
Fabrication steps for multifunctional sensor: (**a**) preparation of auxiliary substrate as a sacrificial layer; (**b**) electrochemical synthesis of PANI NFs as a tensile-sensing material; (**c**) formulation of PDMS for encapsulation; (**d**) etching of ITO glass and applying Cu tape on PANI using silver paste; (**e**) formulation of Ecoflex as dielectric layer; (**f**) overlapping two identical samples in step (**e**) to create a sandwich structure; (**g**) cutting device into a dumbbell shape; (**h**) integration with a photodetector; (**i**) preparation of p-Si substrate; (**j**) hydrothermal synthesis of ZnO NRs as an UV-sensing material; (**k**) deposition of Ag for top electrode; (**l**) bonding Cu tape for bottom electrode. Steps (**a**) through (**g**) are for capacitive sensor using PANI and steps (**i**) through (**l**) are for UV sensor using ZnO.

**Figure 3:**
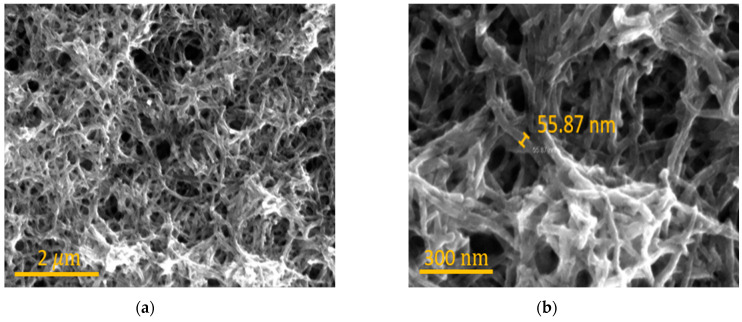
(**a**) SEM image of PANI NFs; (**b**) magnified image showing diameter of PANI NFs.

Figure 2 illustrates the fabrication process for the multifunctional sensor. Initially, the ITO glass was prepared as a sacrificial layer [Figure 2a]. The electrochemical method was employed to synthesize PANI, using ITO glass as the working electrode and a graphite rod as the counter electrode. To deposit PANI NFs, a KPRO-15 (15 μm) photoresist was spin-coated at 1000 rpm for 45 s, followed by patterning using the MA6 lithography equipment (Karl Suss, Munich, Germany). After exposure, the pattern was developed using CD-30 developer for 30 min. Electrodeposition was then carried out at room temperature with a solution of 0.35 M aniline and 0.7 M sulfuric acid at a potential of 1.1 V for 70 s. This process resulted in the formation of a dense PANI layer, which was essential for its later functionality as an electrode [Figure 3a,b]. Following electrodeposition, a lift-off process was used to remove the surrounding photoresist by immersing the sample in acetone for 30 s, leaving only PANI on the ITO glass [Figure 2b]. To encapsulate and remove the sacrificial ITO glass layer, Sylgard 184, mixed with curing agent in a 12:1 ratio, was applied to the PANI layer and spin-coated at 1000 rpm for 30 s. The sample was then cured at 100 °C for 30 min [Figure 2c]. Subsequently, a wet etching process was performed using a hydrochloric acid and deionized water mixture (1:2.3 ratio), which was applied for 10 h at room temperature to fully remove the ITO glass. Next, to create a parallel plate capacitor structure, the encapsulation layers were transferred onto a PEN substrate, ensuring that no air bubbles were trapped. The assembly was then left to dry at room temperature for 24 h. The copper tape was applied to the PANI using silver paste, followed by drying at 100 °C for 10 min and natural drying at room temperature for 6 h [Figure 2d]. Ecoflex was then prepared by mixing Ecoflex00-10 Part A and Part B in a 1:1 ratio. The mixture was spin-coated at 1000 rpm for 30 s to form a 40 μm thick layer [Figure 2e]. The two Ecoflex-PANI layers were then bonded together between two Ecoflex layers to form the capacitive sensor [Figure 2f]. Finally, the tensile sensor was shaped into a dumbbell structure, which is advantageous for tension measurements [Figure 2g] [24].

### 2.5. Fabrication of UV Sensor Using ZnO

**Figure 4:**
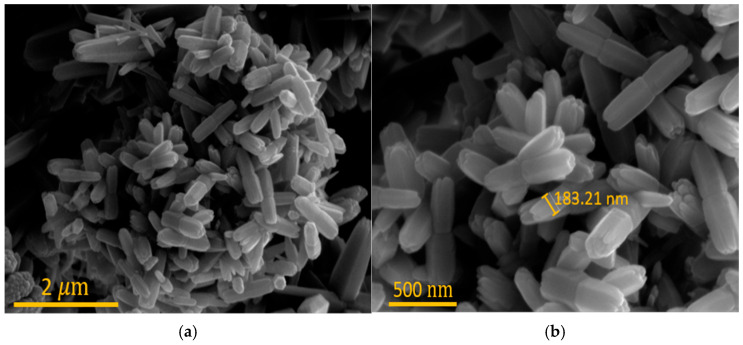
(**a**) SEM image of ZnO NRs; (**b**) magnified image showing diameter of ZnO NRs.

The process begins with the preparation of a p-Si wafer, doped at a concentration of 10^15^ cm^−3^ [Figure 2i]. The wafer was thoroughly cleaned using acetone, buffer oxide etchant, and deionized water. Next, a seed solution was prepared by dissolving 0.02 M zinc acetate dehydrate in 20 mL of n-propanol, followed by sonication for 30 min. This solution was then spin-coated onto the cleaned substrate at 3000 rpm for 30 s and annealed at 100 °C for 6 h. For the hydrothermal growth of ZnO NRs, a solution was prepared by dissolving 4.4 g of zinc nitrate hexahydrate and 2.2 g of methenamine in 300 mL of solvent, and the mixture was stirred at 200 rpm for 30 min. The ZnO NRs were then grown using a microwave-assisted hydrothermal synthesis method [Figure 2j] [32]. After this process, ZnO NRs were grown as shown in Figure 4a,b. This resulted in the formation of a p-Si/n-ZnO pn-heterojunction, which served as the photodetector. To minimize contact resistance, silver electrodes were deposited on the top surface of the ZnO NRs, ensuring an ohmic contact [Figure 2k]. Copper tape was applied to the bottom electrode to secure the ohmic contact [Figure 2l]. The final step involved bonding the fabricated capacitive sensor and photodetector onto a single flexible substrate, completing the multifunctional sensor [Figure 2h].

### 2.6. Stress Performance Calculation

To address the potential risk of photodetector Si substrate fracture under tension, we used finite element modeling (COMSOL Multiphysics, Burlington, MA, USA) to analyze the mechanical stress distribution in the hybrid sensor structure, specifically focusing on the Von Mises stress criterion. Von Mises stress is a key parameter for predicting material yielding under complex loading conditions, making it a reliable tool for assessing the durability of the sensor design [33].

**Figure 5:**
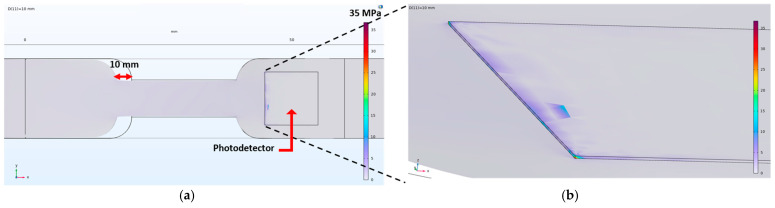
Prediction of Von Mises stress using finite element modeling with 100% strain: (**a**) overall Von Mises stress distribution of sample; (**b**) magnified view of Von Mises stress at edge of Si wafer.

Figure 5a shows the Von Mises stress distribution across the p-Si wafer under 100% elongation, using a sample of dimensions 10,020 μm × 1500 μm. The maximum Von Mises stress occurs at the edge of the wafer, but it remains well below the average fracture stress of Si wafers, which typically ranges from 300 MPa to 3 GPa [34,35]. This indicates that the Si wafer is capable of safely withstanding the applied stress without risk of fracture [Figure 5b].

The simulation results were corroborated by repeatable experimental tests. Even at 100% strain, the Von Mises stress remained significantly below the fracture stress of the Si substrate, with a calculated peak stress of 35 MPa—well within the acceptable range. The substrate can undergo repeated mechanical deformation, confirming that the device maintains both mechanical and electrical stability, even under substantial strain.

## 3. Results

### 3.1. Capacitance Variation in Strain Sensing

**Figure 6:**
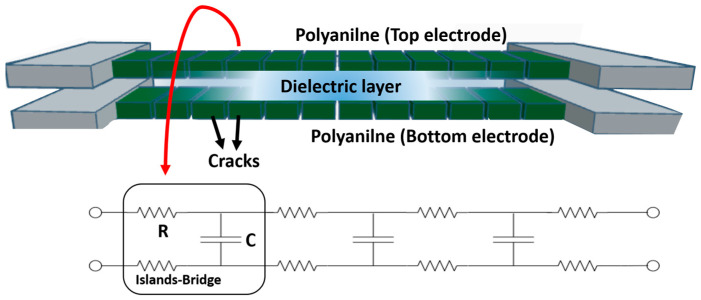
Principle of capacitance generation with PANI used as electrodes, one with a length of 7020 μm and the other with a length of 10,020 μm.

Figure 6 illustrates the principle behind capacitance generation using PANI electrodes. In the absence of strain, capacitance is formed between two parallel electrode plates, as described by the following equation:

(1) $$C=\varepsilon_{0}\varepsilon_{r}A/d$$

where $\varepsilon_{0}$ represents the permittivity of the vacuum, ε*_r_* represents the permittivity of the dielectric layer (Ecoflex), A is the area of the electrode (PANI), and d is the distance between the top and bottom electrodes.

The electrode areas of the sensor are 10.53 mm^2^ (7020 μm × 1500 μm) and 15.03 mm^2^ (10,020 μm × 1500 μm), with a distance d of 40 μm, which allows for the capacitance calculation. When tensile strain is applied, the area increases while the distance decreases, resulting in an overall increase in capacitance. However, because this capacitive sensor uses a conductive polymer as the electrode, its behavior differs from conventional metal electrodes. Under tensile conditions, the PANI forms island-bridges [36], which maintain conductivity but introduce local resistive components on bridges. These components cause charge trapping, which in turn reduces the effective charge density over the larger electrode area, ultimately leading to a decrease in capacitance as the island-bridge structures increase with tensile strain.

**Figure 7:**
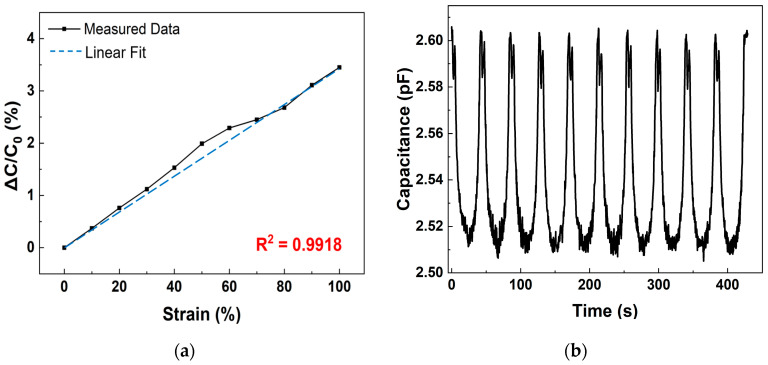

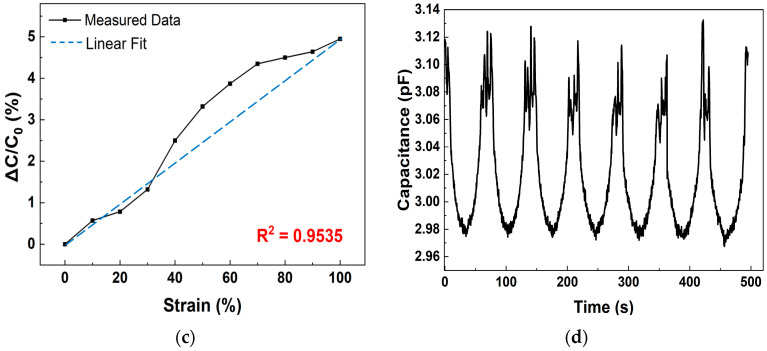
Capacitance variation of multifunctional sensor: (**a**) Δ*C*/*C*_0_ (%) of 7020 μm length sensor; (**b**) transient curve during tensile test of 7020 μm length sensor under 100% strain; (**c**) Δ*C*/*C*_0_ (%) of 10,020 μm length sensor; (**d**) transient curve during tensile test of 10,020 μm length sensor under 100% strain.

This behavior is exploited by the sensor, and the resulting data are shown in Figure 7. The capacitance–time characteristics were measured from 0 to 100% tensile strain in 10% intervals at 1 MHz frequency and 0–2 V AC voltage, using sensors with lengths of 7020 and 10,020 μm, respectively, in an environment maintained at 25 °C and 50% relative humidity. The relative capacitance variation of the strain sensor, Δ*C*/*C*_0_, is calculated using the following equation:

(2) $$\Delta C/C_{0}=\left|C-C_{0}\right|/C_{0}$$

where *C* is the changed capacitance and *C*_0_ is the initial capacitance.

As shown in Figure 7a, the capacitance variation of 7020 μm length sensor increases with tensile strain: 0.37% at 10%, 0.76% at 20%, 1.12% at 30%, 1.53% at 40%, 1.99% at 50%, 2.29% at 60%, 2.45% at 70%, 2.68% at 80%, 3.11% at 90%, and 3.45% at 100%. These values show a clear increase as tensile strain increases, with an R-squared value of 0.9918, indicating excellent linearity.

Figure 7c shows the capacitance variation of the 10,020 μm electrode length sensor. The capacitance variation of the 10,020 μm length sensor also increases with strain, with Δ*C*/*C*_0_ values of 0.5715% at 10%, 0.7851% at 20%, 1.32% at 30%, 2.5% at 40%, 3.32% at 50%, 3.87% at 60%, 4.35% at 70%, 4.5% at 80%, 4.64% at 90%, and 4.95% at 100%. The initial capacitance for the 10,020 μm length sample is 3.14 pF, and the R-squared value of 0.9535 indicates somewhat reduced linearity compared to the 7020 μm length sensor.

**Figure A1:**
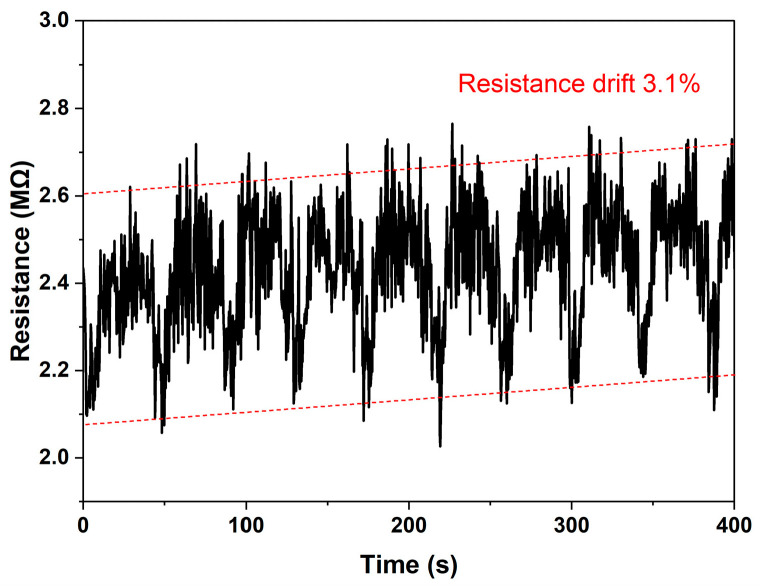
Time-dependent resistance drift during strain sensing from initial to 100% tensile strain.

Figure 7b,d show the transient measurements of capacitance variation over time, with stretching and releasing speed of 10 mm/min and 3 s waiting time under 100% strain. These results demonstrate long-term stability and repeatability, with no significant overshooting or drift as shown in Figure A1. The data highlight the sensor’s strong capacitive sensing capabilities. Additionally, comparing the two results, we observed that as the area of PANI increased, the capacitance value increased by approximately 1.2 times, while an increase in the length of PANI led to reduced stability and a slight increase in hysteresis, due to higher density of PANI island structures and surface non-uniformity.

### 3.2. Capacitance Variation in Pressure Sensing

**Figure 8:**
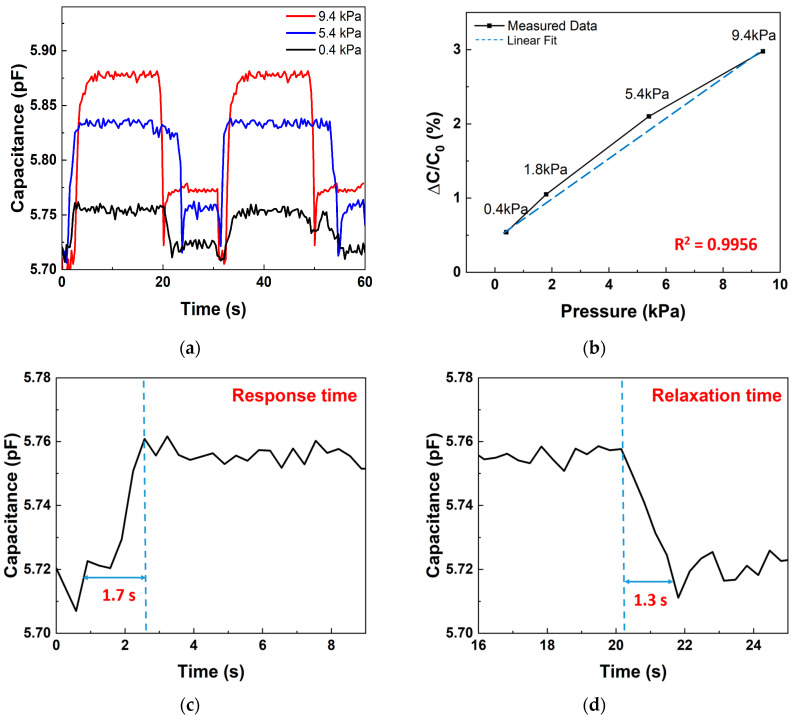
Sensitivity of capacitive pressure sensor: (**a**) capacitance–time transient curves at 0.4 kPa, 1.8 kPa, and 9.4 kPa; (**b**) pressure sensitivity from 0.4 kPa to 9.4 kPa; (**c**) response time at 0.4 kPa; (**d**) relaxation time at 0.4 kPa.

This multifunctional sensor is capable of detecting both strain and pressure. According to the capacitance equation, as pressure increases, the distance d between the PANI electrodes decreases, leading to an increase in capacitance. Figure 8 illustrates the pressure sensing characteristics using a standard 500 mL water bottle. The same frequency and voltage parameters used in the tensile tests were employed. Sequential measurements were taken by increasing the water volume from 0 to 500 mL. The weight of the empty bottle is 13 g (0.4 kPa).

Figure 8a shows the capacitance–time characteristic under 0.4 kPa, 5.4 kPa, and 9.4 kPa. These results demonstrated that capacitance increases as pressure increases. As shown in Figure 8b, the capacitance variation increases with pressure. At 0 mL (0.4 kPa), the capacitance variation was 0.6993%. At 100 mL (1.8 kPa) and 300 mL (5.4 kPa), it increased to 1.051% and 2.101%, respectively, and at 500 mL (9.4 kPa), the variation reached 2.977%. Although a slight hysteresis is inevitably introduced by the viscoelastic recovery of the Ecoflex dielectric layer, the sensor still preserves an excellent linear response (R^2^ = 0.9956) across the entire 0.4 to 9.4 kPa range, confirming its reliability for precise pressure sensing. Moreover, the response time and relaxation time were measured to be 1.7 and 1.3 s, respectively, at the relatively low pressure of 0.4 kPa as shown in Figure 8c,d. These results demonstrate the sensor’s sensitivity to detect small weights, such as the 13 g empty bottle, and its capability to monitor minor pressure changes, making it suitable for applications like human activity monitoring.

### 3.3. Human Monitoring

**Figure 9:**
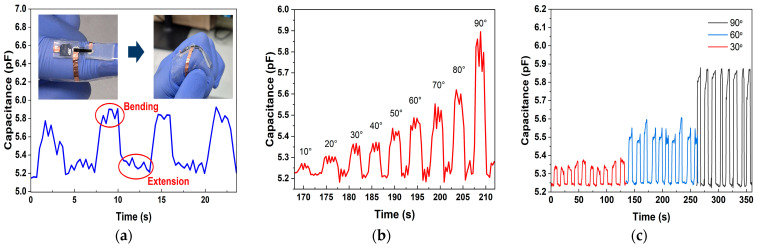
Finger bending monitoring with multifunctional sensor: (**a**) capacitance change during finger bending and extension; (**b**) capacitance–time transient curves at varying finger bending angles from 10° to 90°; (**c**) transient curves of different finger bendings at 30°, 60°, and 90°.

The sensor system was further applied for human activity monitoring, utilizing both strain and pressure sensing capabilities. Figure 9a demonstrates the process of finger bending and extension, showcasing low hysteresis and high stability. Sensitivity as a function of bending angle is presented in Figure 9b, indicating detection of angles ranging from 10° to 90°. Figure 9c presents the transient curves obtained during repeated finger-bending cycles at 30°, 60°, and 90°, which yield error rates of 0.23%, 0.27%, and 0.81%, respectively, thereby confirming the measurement reliability of the proposed sensor.

At a maximum bending angle of 90°, the capacitance variation was observed to be 0.65 pF. This significant variation is attributed to the localized deformation during bending, where fewer PANI islands are formed, minimizing charge trapping. As bending increases, the dielectric layer distance decreases, increasing capacitance consistent with the capacitance equation [Equation (1)]. These findings highlight the system’s potential for monitoring human body movements, paving the way for applications in wearable health monitoring systems.

### 3.4. UV Sensing Through the Photodetector

**Figure 10:**
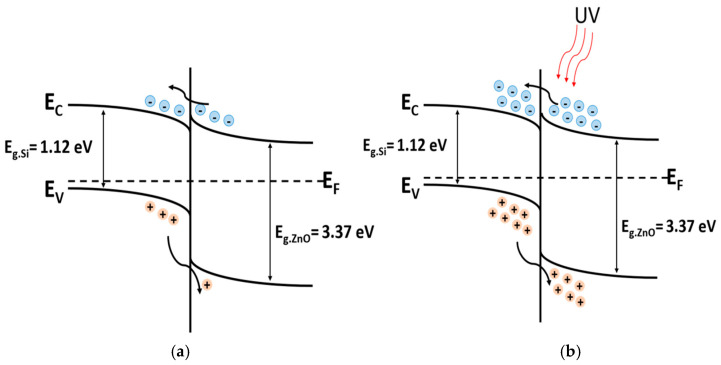
(**a**) Energy band diagram of p-Si/n-ZnO without external stimuli; (**b**) energy band diagram of p-Si/n-ZnO heterojunction under 340 nm UV light.

As illustrated in Figure 1b, the heterojunction photodetector is composed of p-Si and n-ZnO, with a small Ag electrode deposited on top of the ZnO NRs and a copper electrode on the bottom of the p-Si to create ohmic contacts. The energy band diagram in Figure 10a shows the p-Si/n-ZnO heterojunction state without external stimuli, where electrons can easily transfer from ZnO to Si, while holes remain confined within Si due to the energy barrier. In contrast, under UV illumination (Figure 10b), the UV energy generates EHPs, enabling holes to move more freely from Si to ZnO, resulting in enhanced conductivity.

**Figure 11:**
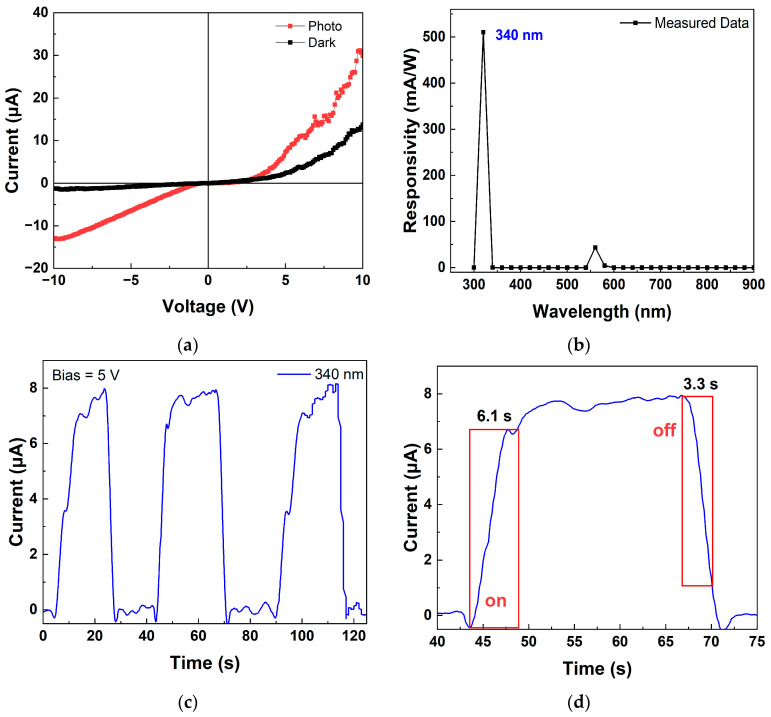
Characteristics of p-Si/n-ZnO UV photodetector: (**a**) current–voltage characteristics under dark and illuminated conditions; (**b**) spectral responsivity of photodetector at 5 V; (**c**) current–time transient response at 340 nm and 5 V; (**d**) response time and relaxation time of photodetector at 340 nm and 5 V.

Figure 11a presents the current–voltage characteristics of the photodetector under dark and UV-illuminated conditions (340 nm) with a voltage sweep from −10 V to 10 V in 0.1 V increments. The device exhibits improved current characteristics under forward bias and significant current flow under reverse bias due to the generation of EHPs. The photodetector also achieves a high on/off ratio of 13.8 at −10 V under reverse bias. In the dark condition, the device exhibits a rectifying ratio of 25.8 at ±10 V, confirming good diode behavior for wearable device.

The spectral responsivity, a critical parameter for assessing a photodetector’s ability to convert incident light into electrical signals, is shown in Figure 11b. Measurements across wavelengths from 300 to 900 nm in 20 nm intervals, 300 W intensity of light, reveal a peak responsivity of 510 mA/W at 340 nm, underscoring the sensor’s high UV sensitivity. The responsivity is calculated using the equation:

(3) $$R=I_{\mathrm{Photo}}-I_{\mathrm{Dark}}/E_{\lambda }S$$

where I_Photo_ is the photocurrent, I_Dark_ is the dark current, $E_{\lambda }$ is the light power intensity, and S is the effective illuminated area.

Transient current measurements at 340 nm UV illumination under a 5 V bias, as shown in Figure 11c, demonstrate low hysteresis and high repeatability. Figure 11d further highlights the photodetector’s rapid response, with a response time of 6.1 s and a recovery time of 3.3 s.

**Figure A2:**
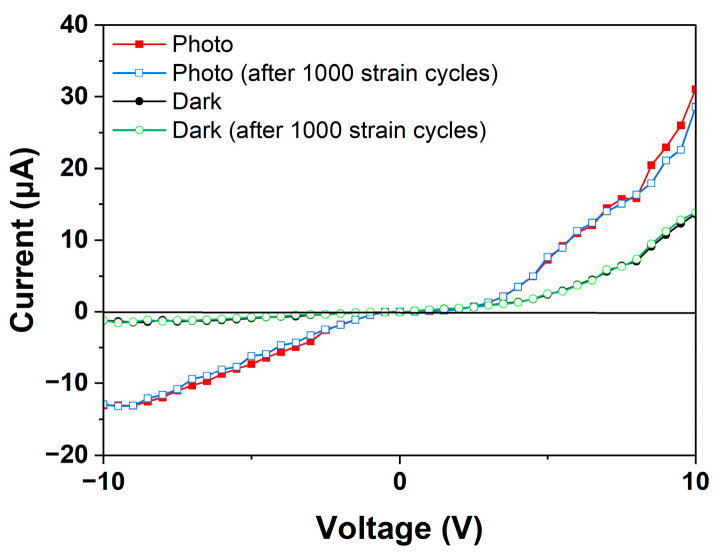
Current–voltage characteristics under dark and photo conditions before and after 1000 cycles at 50% strain.

As shown in Table 1, while ZnO diodes fabricated on rigid and planar substrates routinely achieve sub-second response times and superior diode’s figure of merit, our photodetector offers the fastest response and recovery times reported for a sensor that operates entirely on a flexible substrate. This outstanding performance makes it a strong candidate for wearable multifunctional sensing applications. Moreover, the device maintains electrical characteristics even after 1000 tensile–strain cycles at 50%, underscoring its mechanical robustness and long-term operational stability, as shown in Figure A2.

## 4. Conclusions

This study introduces a multifunctional sensor integrating PANI NFs and ZnO NRs for the simultaneous detections of strain, pressure, and UV light. The sensor combines a capacitive sensing mechanism, utilizing PANI NFs, with a photodetector based on ZnO NRs, all implemented on a single flexible substrate. The capacitive sensor operates using an AC voltage, while the photodetector functions with a DC voltage, enabling distinct AC and DC signal separation for multifunctional sensing. The capacitive sensor demonstrated excellent performance, exhibiting significant capacitance variation even under a maximum strain of 100%, as well as reliable pressure sensing over a broad range. Meanwhile, the photodetector, built on a p-Si/n-ZnO heterojunction, achieved a rapid UV response time of 6.1 s, making it suitable for flexible sensor applications. Finite element modeling revealed an optimal overlap between the flexible substrates and flat Si wafers, contributing to the development of an innovative device that combines the advantages of flexible sensors with the high-performance capabilities of UV photodetectors. This multifunctional sensor also showed efficacy in human activity monitoring, such as detecting finger bending, underscoring its potential for applications in e-skin and wearable devices. Future advancements could enhance sensing performance by incorporating various PANI composites or novel structures. Additionally, the device’s functionality could be expanded to include toxic gas and temperature detection, broadening its potential applications.

## Figures and Tables

**Table 1:** Comparison of various UV sensors using ZnO with respect to on/off ratio, rectifying ratio, response time, and recovery time.

Type	Material	On/Off Ratio	Rectifying Ratio	Response Time	Recovery Time	Reference
Photo Detector	ZnO/Au/Al_2_O_3_	20,000 (Under 0 V)	1000 (Under ±3 V)	<7.4 ms	-	[37]
Photo Detector	Al/ZnO/ITO	1500 (Under 0 V)	-	<156 ms	<319 ms	[38]
Wearable Sensor	ZnO/PDMS	4.2 (Under +5 V)	-	50~60 s	50~60 s	[39]
Wearable Sensor	ZnO/Au(IDT) /PDMS	25.3 (Under +2 V)	-	89 s	100~120 s	[25]
Photo Detector and Wearable Sensor	p-Si/n-ZnO	13.8 (Under −10 V)	25.8 (Under ±10 V)	6.1 s	3.3 s	This Paper

## Data Availability

Data are contained within the article.
